# Positive expression of Y-box binding protein 1 and prognosis in non-small cell lung cancer: a meta-analysis

**DOI:** 10.18632/oncotarget.14732

**Published:** 2017-01-18

**Authors:** Liang Jiang, Gao-Le Yuan, Qi-Lian Liang, Hui-Jie Zhang, Jie Huang, Shao-Ang Cheng, Xiao-Xia Peng

**Affiliations:** ^1^ Oncology Center, Affiliated Hospital of Guangdong Medical University, Zhanjiang, China

**Keywords:** YB-1, NSCLC, IHC, prognosis, meta-analysis

## Abstract

**Background:**

Y-box binding protein 1 (YB-1) belongs to the cold shock domain protein family involved in transcription and translation. We conducted a meta-analysis of the association between YB-1 expression and the survival and clinicopathological features in NSCLC.

**Methods:**

PubMed and Embase were searched to identify studies that evaluated the YB-1 expression (by immunohistochemistry) and overall survival (OS) in NSCLC. Hazard ratios (HRs) and 95% confidence intervals (CI) of OS were pooled. Odds ratios (ORs) of clinicopathological features were computed. Meta-analysis was performed using STATA 12.0 software.

**Results:**

Data on 692 NSCLC patients were collected from six eligible studies. Meta-analysis revealed that YB-1 was associated with worse OS (HR = 1.59, 95% CI [1.27, 2.00], *P* < 0.001, fixed effect), tumor stage (OR = 0.43, 95% CI [0.22-0.82], *P* = 0.01, random effect), and depth of invasion (OR = 0.37, 95%CI [0.22-0.63], *P* < 0.001, fixed effect). A subgroup was analyzed by IHC staining to determine the location of YB-1 positive expression. Poor OS was observed in nucleus staining (pooled HR = 1.86, 95% CI [1.41, 2.45], *P* < 0.001). However, no statistical significance was observed in combined cytoplasmic and nuclear staining (pooled HR = 1.14, 95% CI [0.76, 1.72], *P* = 0.536).

**Conclusions:**

Meta-analysis indicated that YB-1 overexpression is correlated with worse OS and clinicopathological features in NSCLC. Subgroup analysis revealed that the nucleus expression of YB-1 may be more closely associated with NSCLC prognosis than cytoplasmic expression.

## INTRODUCTION

Lung cancer is the most commonly diagnosed cancer and the leading cause of cancer-related deaths among men. It is the second-leading cause of cancer-related deaths among women [1]. The main type of lung cancer is non-small cell lung cancer (NSCLC), which accounts for 85% of all lung cancers, including squamous cell carcinoma, adenocarcinoma, and large-cell carcinoma. In the United States, patients diagnosed with lung cancer manifest a five-year survival rate of 16.5% [2]. Such rates are even lower in developing countries. YB-1 was recently reported as a poor prognosis of many malignant tumors, and its role in NSCLC caught our interest.

Y-box binding protein 1 (YB-1) is a multifunction protein belonging to the cold shock domain protein family that binds both DNA and RNA [3]. As a role of transcription factor, YB-1 binds to the Y-box (inverted CCAAT box) [4]. Moreover, as a component of the messenger ribonucleoprotein particle mRNP complex, YB-1 regulates the translational activity of mRNP [5–7]. YB-1 participates in almost all DNA and mRNA related processes in the cell [8], and it is involved in therapy resistance, gene splicing/repair, exon skipping, and cancer development [9]. YB-1 is a multifunction protein that enhances the ten cancer hallmarks proposed by Hanahan and Weinberg [9, 10]. Given its multifunction, YB-1 was considered a therapeutic target. The results showed that anti-YB-1 siRNAs can suppress tumor cell proliferation, invasion, and differentiation; it also promotes apoptosis and enhances chemosensitivity [11–15]. In the clinic, YB-1 was detected in the immunohistochemical staining of the nucleus and/or the cytoplasm. Recent studies found that YB-1 overexpression is involved in several malignant tumors, such as breast cancer [16], colorectal carcinoma [17, 18], glioma [19], renal cell carcinomas [20], prostate cancer [21], and lung cancer [22].

Although many studies have reported on YB-1, the prognostic value of YB-1 overexpression in NSCLC remains unclear. Thus, meta-analysis should be performed to evaluate whether YB-1 overexpression is associated with poor outcome. The results of the meta-analysis will serve as basis for the development of therapeutic targets for this receptor.

## RESULTS

### Description of studies

**Figure 1:**
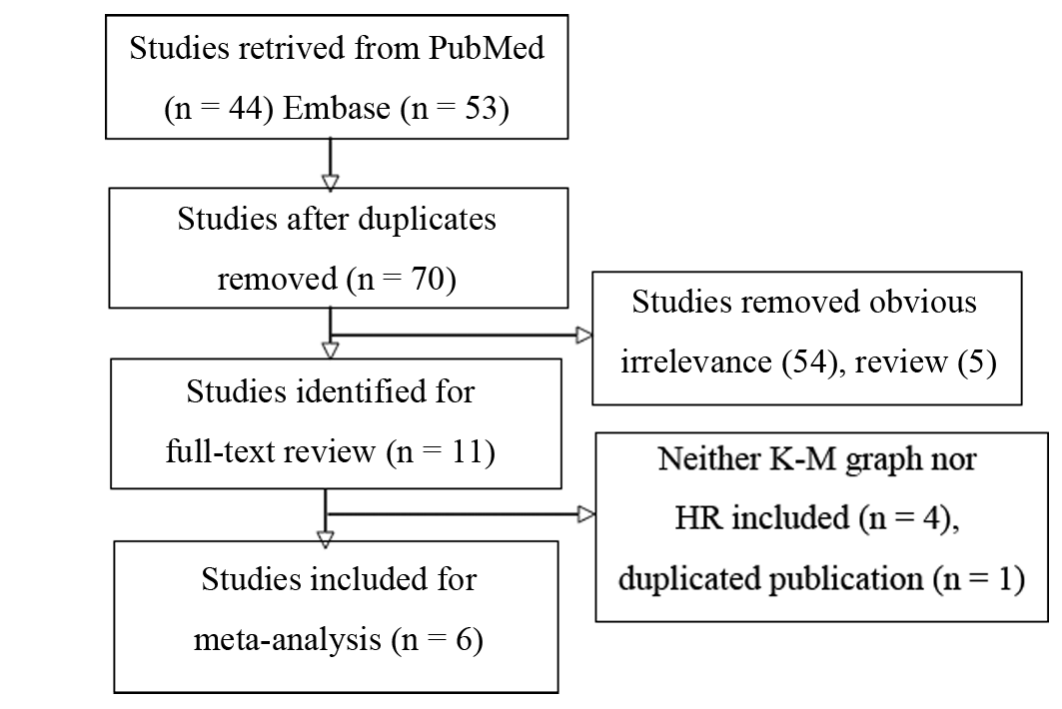
Flow chart for selection of studies

We indentified six studies (Figure 1) that analyzed the prognostic value of YB-1 expression [22–27]. The characteristics of these studies are shown in Table 1. Out of the six studies, four evaluated patient cohorts from Japan, and one each evaluated patients from China and Germany. These studies gathered 692 NSCLC patients. The recruitment time in five studies (576 patients) was before 2005, and that in the remaining study (116 patients) was between 2008 and 2010. Three studies reported hazard ratios (HRs) and their 95% confidence intervals (CI). The other three studies extracted Kaplan-Meier curves. All selected articles were retrospective cohort studies and were of high quality according to the Newcastle Ottawa scale (NOS) quality criteria.

**Table 1:** Main characteristic of the six selected studies in this meta-analysis

Study(author-reference-year)	country	Recruitment time	No. of patients	Time of follow-up(month)	Analysis of variance	HR estimation	NOS
Zhao et al. [22](2016)	China	2008-2010	116	3-60	Multivariate	1.21(0.50-2.95)^a^	6
Yoshimatsu et al. [23](2005)	Japan	1979-1987	94	18.3m	Univariate	1.12( 0.70-1.77)^a^	7
Shibahara et al. [24](2001)	Japan	1990-1994	196	25-110	Multivariate	1.47(0.93-2.32)^b^	7
Kashihara et al. [25](2009)	Japan	1997-2004	104	5-126	Univariate	1.73(1.05-2.83)^a^	6
Hyogotani et al. [26](2012)	Japan	2000-2001	105	NR	Univariate	3.44(1.24-9.53)^b^	5
Gessner et al. [27](2004)	Germany	1991-1996	77	NR	Multivariate	2.37(1.36-4.10)^b^	5

NR data were not reported. NOS Newcastle Ottawa scale (NOS) quality criteria.

^m^ median follow-up time.

^a^ directly extracted from original data.

^b^ extrapolated from Kaplan-Meier curves.

### Evaluation and expression of YB-1

Table 2 presents the details of immunohistochemistry (IHC). The IHC positive rate value ranged from 37.1% to 48.1%. Various antibodies and antibody epitopes were used to evaluate YB-1 expression. The cutoff for overexpression depended on the staining location and the selected scored method. Two studies used a combined cytoplasmic and nuclear staining to determine the positive status [22, 23]. Four studies used nucleus staining only [24–27].

**Table 2:** Evaluation of YB-1 by IHC in the six selected studies

Study (reference)	yb-1 positive rate	Antibody epitope	Cutoff for positive
Zhao et al. [22]	37.1%	NI	Cytoplasm, nucleus; Final score were the product of the score for staining intensity (range 0 to 3) and staining cell numbers [range 1 to 4:1(0-25%); 2(26-50%); 3(51-75%); 4(> 75%)]. Positive: ≥6
Yoshimatsu et al. [23]	45.7%	AA 299-313	Cytoplasm, nucleus; positive: >60% of the tumor cell nucleus or cytoplasm stained
Shibahara et al. [24]	44.9%	AA 299-313	Cytoplasm, nucleus; positive: nucleus stained
Kashihara et al. [25]	42.3%	AA 299-313	Cytoplasm, nucleus; positive: nucleus strong expression
Hyogotani et al. [26]	38.1%	AA 299-313	Nucleus; Three microscopic fields from the greatest accumulation of positive signals (hotspots) were selected. The mean values of percentage of stained cells were calculated; positive: >10%
Gessner et al. [27]	48.1%	AA 1-12	Nucleus; Four microscopic fields (400x) were selected, percentage of moderate or intense nuclear stained cells (0–100% in 5% steps) were calculated; positive: >10%

AA amino acid.

NI not indicated.

### Association of YB-1 with Survival

**Figure 2:**
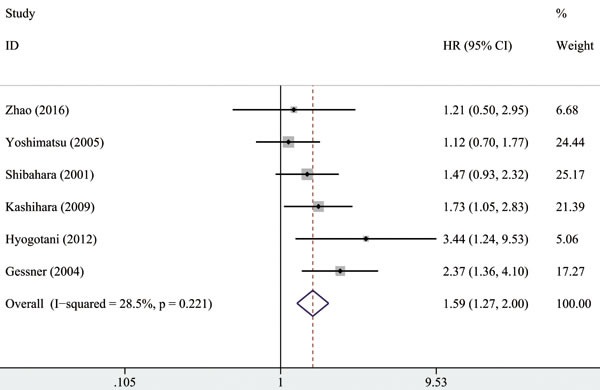
Forest plot showing the HR of YB-1 overexpression *vs*. normal YB-1 expression for OS The weight of each study in the meta-analysis is illustrated by the size of squares, and the extending line crossing the square illustrates the 95% CI. The diamond represents the pooled HR performed by the fixed-effect model.

Pooled analysis of the six studies showed that YB-1 overexpression was associated with the worst overall survival (OS) in NSCLC patients (HR = 1.59, 95% CI [1.27, 2.00], *P* < 0.001, fixed effect). A moderate heterogeneity (*I*^2^ = 28.5%, *P*_H_ = 0.221) was observed (Figure 2 and Table 3). Table 3 shows the results of the subgroup meta-analyses. All pooled HRs were obtained by using a fixed-effect model. Results showed in terms of country, unfavorable prognosis was found in Japan (pooled HR = 1.49, 95% CI [1.15, 1.94], *P* = 0.003). Poor prognosis also was found in NSCLC with YB-1 overexpression under univariate analyses (pooled HR = 1.50, 95% CI [1.09, 2.07], *P* = 0.013) and multivariate analyses (pooled HR = 1.69, 95% CI [1.22, 2.35], *P* = 0.002). When subgrouped by the IHC staining location of YB-1 overexpression, poor OS was observed in nucleus staining (pooled HR = 1.86, 95% CI [1.41, 2.45], *P* < 0.001), whereas no statistical significance was found in combined cytoplasmic and nuclear staining (pooled HR = 1.14, 95% CI [0.76, 1.72], *P* = 0.536). When the study with the latest recruitment time was excluded, an unfavorable survival result was obtained (HR = 1.63, 95%CI [1.28, 2.06], *P* < 0.001).

**Table 3:** Meta-analysis of YB-1 overexpression and prognosis in NSCLC

Categories	Studies (patients)	HR (95%CI)	*I*^2^ (%)	*P*_H_	Z	*P*
Overall survival	6(692)	1.59(1.27-2.00)	28.5	0.221	3.98	<0.001
Country (Japan)	4(499)	1.49(1.15-1.94)	31.7	0.222	2.98	0.003
Univariate analyses	3(303)	1.50(1.09-2.07)	54.4	0.112	2.49	0.013
Multivariate analyses	3(389)	1.69(1.22-2.35)	14.7	0.310	3.16	0.002
Nucleus staining	4(481)	1.86(1.41-2.45)	7.3	0.356	4.38	<0.001
Cytoplasm, nucleus staining	2(210)	1.14(0.76-1.72)	0	0.880	0.62	0.536
Recruitment time (before 2005)	5(576)	1.63(1.28-2.06)	39.3	0.159	4.01	<0.001

All pooled HRs were performed by fixed-effect model.

*P*_H_ *P*-value for heterogeneity based on Q test.

*P* *P*-value for statistical significance based on Z test.

### Association of YB-1 with clinicopathological parameters

**Figure 3:**
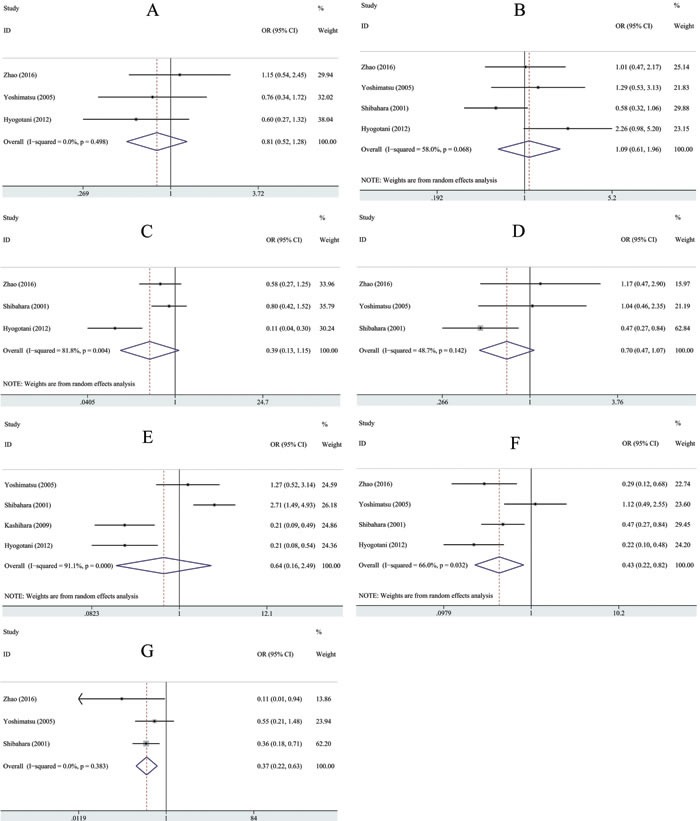
Forest plots showing the OR of YB-1 overexpression *vs*. normal YB-1 expression for clinicopathological features **A**. Age **B**. Sex **C**. Tumor differentiation **D**. Lymph node metastasis **E**. Histology **F**. Tumor stage **G**. Depth of invasion. All pooled ORs were obtained by using a fixed-effect model except for figure marked with (NOTE: Weights are from random effects analysis).

The associations between YB-1 and clinicopathological parameters are shown in Table 4 and Figure 3. The differences between YB-1 overexpression and biologically aggressive phenotypes, such as tumor stage (OR = 0.43, 95% CI [0.22-0.82], *P* = 0.01, random effect) and depth of invasion (OR = 0.37, 95%CI [0.22-0.63], *P* < 0.001, fixed effect), were statistically significant. However, no association was found between YB-1 and other clinicopathological features, including age (OR = 0.81, 95%CI [0.52-1.28], *P* = 0.37, fixed effect), sex (OR = 1.09, 95% CI [0.61-1.96], *P* = 0.77) tumor differentiation (OR = 0.39, 95% CI [0.14-1.15], *P* = 0.09, random effect), lymph node metastasis (OR = 0.71, 95% CI [0.47-1.07], *P* = 0.1, fixed effect), and histology type (OR = 0.64, 95% CI [0.16-2.49], *P* = 0.52, random effect).

**Table 4:** Meta-analysis of YB-1 overexpression and clinicopathological features in NSCLC

Categories	Studies (patients)	OR (95%CI)	*I*^2^ (%)	*P*_H_	Z	*P*
Age (≤64 / >64)	[22, 23, 26] (315)	0.81 (0.52-1.28)	0.0	0.498	0.89	0.37
Sex (Male / Female)	[22–24, 26] (511)	1.09(0.61-1.96)R	58.0	0.068	0.29	0.77
Tumor differentiation (Well / Morate, Poor)	[22, 24, 26] (408)	0.39(0.14-1.15)R	81.8	0.004	1.70	0.09
Lymph node metastasis (Absent / Present)	[22–24] (406)	0.71(0.47-1.07)	48.7	0.142	1.65	0.10
Histology( adenocarcinoma / squamous cell carcinoma)	[23–26] (485)	0.64(0.16-2.49)R	91.1	0.000	0.64	0.52
Tumor stage ( I / II,III,IV)	[23–24, 26] (521)	0.43(0.22-0.82)R	66.0	0.032	2.56	0.01
Depth of invasion (T1-T1/T3-T4)	[22–24] (406)	0.37(0.22-0.63)	0	0.383	3.63	<0.001

All pooled ORs were performed by fixed-effect model except for cells marked with (randomR).

*P*_H_ *P*-value for heterogeneity based on Q test.

*P* *P*-value for statistical significance based on Z test.

### Sensitivity analysis and publication bias

**Figure 4:**
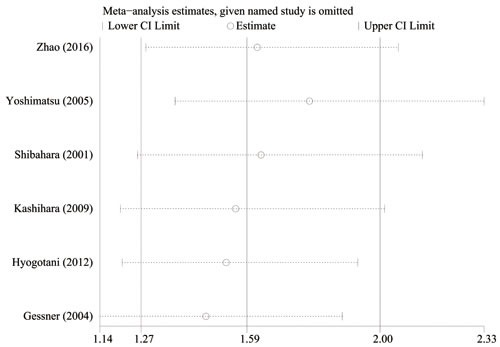
Effect of individual studies on the pooled HR forYB-1 overexpression and OS of NSCLC The horizontal axis number 1.59 represents the overall HR, and the 1.27 and 2.00 represent the 95% CI.

The results of the sensitivity analysis are shown in Figure 4. When each individual study was sequentially excluded, the combined 95% CI of the remaining five studies did not exceed the 95% CI of the pooled HR of six studies, indicating that no individual study dominated the results. Publication bias was tested by HR estimation of the OS. No obvious publication bias was revealed by Egger's tests (*t* = 0.97, *P* = 0.388, 95% CI [−3.2, 6.7]), Begg's test (*Z* = 0.75, *P* = 0.452), and visual inspection of funnel plots.

## DISCUSSION

In this study, we meta-analyzed the literature on YB-1 expression in NSCLC and its association with OS and clinicopathological features. Results showed that YB-1 overexpression was correlated with poor OS. All subgroup and sensitivity analyses indicated the poor role of YB-1 overexpression in NSCLC except for the combined cytoplasmic and nuclear staining [22, 23]. When subgroup analyses in terms of the IHC staining location of YB-1 overexpression were used, heterogeneity was significantly reduced. The cutoff for the positive value considered only the nucleus staining that showed a statistical significance [24–27], suggesting that only the nucleus expression of YB-1 was associated with poor OS in NSCLC. Moreover, multivariate analyses in the subgroup analyses showed a statistical significance [22, 24, 27]. Thus, YB-1 expression may be an independent factor of OS. We also found a significant association between YB-1 overexpression and poor clinicopathological features, including tumor stage and depth of invasion. Although this study is a literature-based analysis, Begg's test, Egger's test, and funnel plot found no publication bias. Heterogeneity assumption was tested by *I*^2^ metric, and the pooled HR of OS displayed moderate heterogeneity. These results are encouraging and serve as basis for the further development of biomarkers and target therapy.

This study has a number of limitations. First, various antibodies and epitopes of the YB-1 antibody may lead to different nucleo-cytoplasmic staining results. Second, no method and cutoff definition have been accepted and validated for evaluating YB-1 expression. Third, only a small number of patients were considered in this study. Finally, we failed to directly extract the HRs and 95% CI from the original data of three selected studies. Instead, we estimated them from the survival curves. Although estimating the estimated HRs may be a less reliable approach, it was the only feasible method [28]. We analyzed the pooled HRs by using two ways of HR extraction, and we found no major deviations.

In conclusion, this meta-analysis showed that YB-1 overexpression is correlated with poor OS and clinicopathological features in NSCLC, suggesting that YB-1 may be a poor prognostic factor and therapeutic target in NSCLC. Subgroup analysis revealed that the nucleus expression of YB-1 may be more closely associated with the prognosis of NSCLC than cytoplasm expression. Large well-designed studies employing a standard evaluated method are necessary to obtain higher-quality evidence.

## MATERIALS AND METHODS

Meta-analysis was conducted in accordance with the preferred reporting items for meta-analysis criteria [29].

### Search strategy

We searched PubMed and Embase from inception up to July 18, 2016. The following search strategies were used for search PubMed: (‘Y-Box-Binding Protein 1’ [Mesh] OR ‘Y-Box-Binding Protein 1’ OR ‘yb-1’ OR ‘yb1’ OR ‘ybx1’ OR ‘ybx-1’) AND (‘Lung Neoplasms’ [Mesh]) OR ‘lung neoplasms’ OR ‘lung cancer’ OR ‘lung tumor’ OR ‘lung carcinoma’); and Embase: (‘Y-Box-Binding Protein 1’/exp OR ‘Y-Box-Binding Protein 1’ OR ‘yb 1’ OR ‘yb1’ OR ‘ybx 1’ OR ‘ybx1’) AND (‘Lung Cancer’/exp OR ‘lung cancer’ OR ‘Lung Tumor’/exp OR ‘lung tumor’ OR ‘lung carcinoma’).

### Selection of studies

The selected studies should meet the following eligibility criteria: (1) retrospective or prospective cohort studies; (2) published in English; (3) based on the association between YB-1 and NSCLC; (4) availability of OS data for estimating HR and 95% CI; and (5) YB-1 expression was measured by IHC. When duplicate studies or overlapping patient cohorts were found, only the largest or most informative data were included. Reviews, letters, editorials, unpublished studies, and conference abstracts were excluded.

### Data extraction

Two reviewers (Jiang L and Yuan GL) independently extracted information with the use of a predesigned Excel sheet. The following information were extracted: name of the first author, publication time, country, number of patients, recruitment time, follow-up duration, analysis method, tumor type, clinicopathological features, antibody epitope, method and score for its evaluation, cutoff for considering YB-1 overexpression, positive rate, HR, and their 95% CI. If HR and their 95% CI were not reported, we extracted them from Kaplan-Meier curves by using the methods proposed by Tierney et al. [28].

### Quality assessment

The NOS was used to assess the quality of the selected studies. The NOS included three main aspects: selection, comparability, and outcome [30, 31]. A study with a score of at least 5 was considered of high quality.

### Statistical analysis

Meta-analysis was performed using STATA software (version 12.0; StataCorp, College Station, Texas, USA). Pooled HRs of OS and their 95% CI were calculated. Subgroup meta-analyses, which determined the possible factors that may influence the results, were conducted for: (1) country of patient cohorts; (2) ANOVA; (3) IHC staining location of YB-1 overexpression; and (4) patient recruitment time. The relative frequency of the correlation between YB-1 overexpression and clinicopathological features (age, sex, tumor differentiation, lymph node metastasis, histology, tumor stage, and depth of invasion) was expressed as OR and its 95% CI.

Heterogeneity assumption was tested using the chi-squared test based on the Q statistic [32]. If *PH* < 0.10 revealed significant heterogeneity, the pooled HR or OR were obtained by using a random-effect model. Otherwise, a fixed-effect model was used. We also quantified the heterogeneity by *I*^2^ metric (*I*^2^ < 25%, 25%≤ *I*^2^≥50%, *I*^2^ > 50%, represent low, moderate, and extreme heterogeneity, respectively) [32]. Sensitivity analysis was tested by using the “metaninf” STATA command (sequential exclusion of each individual study then pooled HR). Visual inspection of funnel plots, Begg's test, and Egger's asymmetry tests [33] were used to evaluate publication bias (*P* < 0.10 was considered statistically significant).
